# Comparative Proteomics Reveals Differences in Host-Pathogen Interaction between Infectious and Commensal Relationship with *Campylobacter jejuni*

**DOI:** 10.3389/fcimb.2017.00145

**Published:** 2017-04-26

**Authors:** Nieves Ayllón, Ángeles Jiménez-Marín, Héctor Argüello, Sara Zaldívar-López, Margarita Villar, Carmen Aguilar, Angela Moreno, José De La Fuente, Juan J. Garrido

**Affiliations:** ^1^SaBio, Instituto de Investigación en Recursos Cinegéticos (CSIC-UCLM-JCCM)Ciudad Real, Spain; ^2^Grupo de Genómica y Mejora Animal, Departamento de Genética, Facultad de Veterinaria, Universidad de CórdobaCórdoba, Spain; ^3^Department of Veterinary Pathobiology, Center for Veterinary Health Sciences, Oklahoma State UniversityStillwater, OK, USA

**Keywords:** human, pig, intestinal epithelial cells, SWATH-MS, immunity, infection

## Abstract

*Campylobacter jejuni* is the leading food-borne poisoning in industrialized countries. While the bacteria causes disease in humans, it merely colonizes the gut in poultry or pigs, where seems to establish a commensal relationship. Until now, few studies have been conducted to elucidate the relationship between *C. jejuni* and its different hosts. In this work, a comparative proteomics approach was used to identify the underlying mechanisms involved in the divergent outcome following *C. jejuni* infection in human and porcine host. Human (INT-407) and porcine (IPEC-1) intestinal cell lines were infected by *C. jejuni* for 3 h (T3h) and 24 h (T24h). *C. jejuni* infection prompted an intense inflammatory response at T3h in human intestinal cells, mainly characterized by expression of proteins involved in cell spreading, cell migration and promotion of reactive oxygen species (ROS). Proteomic analysis evidenced significantly regulated biofunctions in human cells related with engulfment and endocytosis, and supported by canonical pathways associated to infection such as caveolar- and clathrin-mediated endocytosis signaling. In porcine IPEC-1 cells, inflammatory response as well as signaling pathways that control cellular functions such as cell migration, endocytosis and cell cycle progression resulted downregulated. These differences in the host response to infection were supported by the different pattern of adhesion and invasion proteins expressed by *C. jejuni* in human and porcine cells. No marked differences in expression of virulence factors involved in adaptive response and iron acquisition functions were observed. Therefore, the results of this study suggest that both host and pathogen factors are responsible for commensal or infectious character of *C. jejuni* in different hosts.

## Introduction

Human campylobacteriosis is leading the rank of food-borne diseases in developed countries, with *Campylobacter* being the most regularly reported zoonotic pathogen in the European Union since 2005 (EFSA, 2015). The disease is usually associated with self-limiting mild diarrhea, which in particular cases such as immunocompromised patients can progress to a severe systemic infection (Clark et al., 2016). In addition to diarrhea, campylobacteriosis has been linked to inflammatory bowel disease (Spiller, 2007) and reactive arthritis or Guillain-Barré syndrome (Yuki et al., 2004).

Human *Campylobacter* infections are caused mainly by species *Campylobacter jejuni* and *C. coli*, although the former accounts for more than 90% of the human infections (Wilson et al., 2008). Several environmental and animal reservoirs may become the origin of human infections (Young et al., 2007), although the primary source of human campylobacteriosis is contaminated chicken and pork meat (Wilson et al., 2008). *Campylobacter* spp. usually colonizes the gut of chickens and pigs, where it seems to establish a commensal relationship, harboring concentrations of 10^7^–10^10^ CFU/gram of feces in their intestines but lacking the development of clinical signs in the host (Battersby et al., 2016). Despite the importance of the disease in humans, our understanding of the virulence mechanisms of *Campylobacter* is still relatively poor (Dasti et al., 2010; Aguilar et al., 2014). Moreover, little is known about the mechanisms that determine the pathogenic of commensal character of *Campylobacter* in different hosts. In a previous *in vitro* study, we revealed that *C. jejuni* preferentially interacted with human intestinal epithelial cells in which the level of bacterial invasion was up to 10-fold higher than in porcine intestinal cells (Aguilar et al., 2014). This study also showed that a strong inflammatory response occurred in human cells after bacterial infection while no response was observed in intestinal epithelial cells of porcine origin. Despite these results, much is still unknown about why *Campylobacter* is pathogenic to humans and not to other species such as pigs. Here, we surveyed the proteome of human and porcine intestinal epithelial cells after *C. jejuni* infection in order to elucidate the molecular mechanisms underlying the pathological or commensal behavior of *Campylobacter* in different hosts.

## Materials and methods

### Cell lines and culture conditions

Porcine intestinal epithelial cell line IPEC-1, derived from the small intestine of a newborn unsuckled piglet, was cultured in Dulbecco's Modified Eagle Medium (DMEM)/Ham's F-12 (1:1) medium (Life Technologies, Carlsbad, CA, USA) supplemented with 5% fetal bovine serum (FBS, PAA Laboratories GmbH, Pasching, Austria), epidermal growth factor (5 μg/mL) (Sigma–Aldrich, St. Louis, MO, USA), insulin (10 μg/mL), transferrin (5 μg/mL), sodium selenite (5 ng/mL) (ITS Premix, Sigma) and 2 mM L-glutamine (Life Technologies). Human intestinal epithelial cells INT-407 (human embryonic intestine, ATCC CCL-6) were cultured in RPMI-1640 medium (Lonza, Basel, Switzerland) and supplemented with 10% FBS (PAA Laboratories GmbH) and 2 mM L-glutamine (Life Technologies). All cell lines were seeded in multi-well tissue culture plates (Thermo Fischer Scientific, Waltham, USA) the day before the assay, and allowed to reach confluence for the *in vitro* infection. The cells were maintained in an atmosphere of 5% CO_2_ at 37°C.

### Bacterial strain and *In vitro* infection

A confirmed isolate of *C. jejuni* from chicken feces was recovered from stocks kept at −80°C by plating on Columbia sheep blood agar (Oxoid, Basinstoke, Hampshire, UK) for 48 h at 37°C under microaerobic conditions (AnaeroGen system, Oxoid). The bacteria were harvested from plate and resuspended in fresh cell culture media. The optical density (OD_600_) was adjusted to 1 to achieve 10^8^ CFU/ml for their straight inoculation into INT-407 and IPEC-1 cells at a multiplicity of infection (MOI) of 100/1. All *in vitro* cell infections with bacteria were performed in triplicate as previously described (Aguilar et al., 2014) using two points in the study time course: 3 h (early time, T3h) and 24 h (late time, T24h). The same time course was used with the uninfected controls.

### Protein extraction and trypsin digestion

The cells were centrifuged at 10,000 × g for 3 min, and cell pellets were frozen in liquid nitrogen until used for protein extraction. Approximately 10^7^ cells were pooled from each time point and homogenized with a needle (27G) in 500 μl lysis buffer [1% Triton X-100 supplemented with Complete protease inhibitor cocktail (Roche, Basel, Switzerland)]. The samples were sonicated for 1 min in an ultrasonic cooled bath, followed by 10 s of vortexing. After 3 cycles of sonication-vortexing, total cell extracts were centrifuged at 200 × g for 5 min to remove cell debris. The supernatants were collected and protein concentration was determined using the Bradford Protein Assay (Bio-Rad, Hercules, CA, USA) with bovine serum albumin (BSA) as standard. Protein extracts (150 μg) were on-gel concentrated by SDS-PAGE as previously described (Villar et al., 2014). The unseparated protein bands were visualized by staining with GelCode Blue Stain Reagent (Thermo Scientific, Waltham, MA, USA), excised, cut into 2 × 2 mm cubes and digested overnight at 37°C with 60 ng/ml of sequencing grade trypsin (Promega, Madison, WI, USA) at 5:1 protein:trypsin (w/w) ratio in 50 mM ammonium bicarbonate, pH 8.8 containing 10% (v/v) acetonitrile (Shevchenko et al., 2006). The resulting tryptic peptides were extracted by 30 min-incubation in 12 mM ammonium bicarbonate, pH 8.8. Trifluoroacetic acid was added to a final concentration of 1% and the peptides were finally desalted onto OMIX Pipette tips C18 (Agilent Technologies, Santa Clara, CA, USA), dried-down and stored at –20°C until mass spectrometry analysis.

### Proteome analysis by SWATH-MS

The desalted protein digest was resuspended in 5% acetonitrile with 0.1% formic acid and analyzed by reverse phase liquid chromatography coupled online with mass spectrometry (RP-LC-MS/MS) using an Ekspert nLC 415 system coupled to a 6,600 TripleTOF mass spectrometer (AB SCIEX, Framingham, US) through Information-Dependent Acquisition (IDA) followed by SWATH (Sequential Windowed data independent Acquisition of the Total High-resolution Mass Spectra). Approximately 4 μg of each protein digest from each of the replicate samples were pooled together as a mixed sample for each condition (control and infected cells) and each cellular type. Pooled mixed samples were then used for the generation of the reference spectral ion library as part of SWATH-MS analysis. The peptides were concentrated using a 0.1 × 20 mm C18 RP precolumn (Thermo Scientific), and then separated using a 0.075 × 250 mm C18 RP column (New Objetive, Woburn, MA, USA) operating at 0.3 ml/min. Peptides were eluted using a 120-min gradient from 5 to 40% solvent B followed by 15-min gradient from 40 to 60% solvent B (Solvent A: 0.1% formic acid in water, solvent B: 0,1% formic acid in acetonitrile) and directly injected into the mass spectrometer for analysis. Three technical replicates of each mixed sample were analyzed. For IDA experiments, the mass spectrometer was set to scanning full spectra (350–1,400 m/z) using 250 ms accumulation time per spectrum, followed by up to 50 MS/MS scans (100–1,500 m/z). Candidate ions with a charge state between +2 and +5, and counts per second above a minimum threshold of 100, were isolated for fragmentation. One MS/MS spectrum was collected for 100 ms, before adding those precursor ions to the exclusion list for 15 s (mass spectrometer operated by Analyst® TF 1.6, AB SCIEX). Dynamic background subtraction was turned off. MS/MS analyses were recorded in high sensitivity mode with rolling collision energy on and a collision energy spread of 5. For SWATH quantitative analysis, 8 μg of each mixed sample were subjected in triplicate to the cyclic data independent acquisition (DIA) of mass spectra using the SWATH variable windows calculator (V 1.0, AB SCIEX) and the SWATH acquisition method editor (AB SCIEX), following previously established methods (Gillet et al., 2012). A set of 50 overlapping windows was constructed (containing 1 m/z for the window overlap), covering the precursor mass range of 400–1,250 m/z. For these experiments, a 50 ms survey scan (350–1,400 m/z) was acquired at the beginning of each cycle, and SWATH-MS/MS spectra were collected from 100 to 1,500 m/z for 70 ms at high sensitivity mode, resulting in a cycle time of 3.6 s. Collision energy for each window was determined according to the calculation for a charge +2 ion-centered upon the window with a collision energy spread of 15.

### Library generation/protein identification, data processing, and relative quantitation

To create a spectral library with those peptides that have been detected and identified in the experimental time course, the IDA MS raw files were combined and subjected to database searches in unison using ProteinPilot software v. 5.0.1 (AB SCIEX) with the Paragon algorithm. Spectra identification was performed by searching against the *Homo sapiens* proteome for INT samples, Sus scrofa taxa for IPEC-1 samples and the *C. jejuni* taxa for INT and IPEC infected samples (Uniprot Databases: 70,826, 34,409, and 41,050 entries respectively, in December 2015) with the following parameters: iodoacetamide cysteine alkylation, trypsin digestion, gel-based ID as special factor, identification focus on biological modification and evolutionary variants and thorough ID as search effort. The detected protein threshold was set at 0.05. An independent False Discovery Rate (FDR) analysis, using the target- decoy approach provided by ProteinPilot, was used to assess the quality of identifications. Positive identifications were considered when identified proteins reached a 1% global FDR. For SWATH processing, up to 10 peptides with seven transitions per protein were automatically selected by the SWATH Acquisition MicroApp2.0 in the PeakView2.2 software (AB SCIEX) with the following parameters: 15 ppm ion library tolerance, 5 min XIC extraction window, 0.01 Da XIC width, and considering only peptides with at least 99% confidence and excluding those which were shared or contained modifications. However, to ensure reliable quantitation, only proteins with 3 or more peptides available for quantitation were selected for XIC peak area extraction and exported for analysis in the MarkerView 1.3 software (AB SCIEX). Global normalization was performed according to the Total Area Sums of all detected proteins in the samples. A Student's *T*-test was used to perform two-sample comparisons between the averaged area sums of all the transitions derived for each protein across the three replicate runs for each sample under comparison, in order to identify proteins that were significantly differentially represented between infected and uninfected samples.

### Systems biology analysis

Ingenuity Pathway Analysis (IPA) web-based application (Ingenuity Systems Inc., Redwood City, CA, USA) was used to assess the biological meaning in the host proteome datasets. IPA retrieves biological information from the literature and then integrates the differentially expressed proteins into functions and pathways with biological meaning and significance (*p* < 0.05). Functional association networks of the potentially interacting proteins were generated using STRING (Search Tool for the Retrieval of Interacting Genes, v.10 web server, http://stringdb.org/), a database of known and predicted protein interactions (Szklarczyk et al., 2011). Virulence factors expressed in *C. jejuni*-infected INT-407 cells were used as dataset framework for mapping functional pathways. The model was enriched up to 100 partners with different sorts of associations to the proteins included in the model.

## Results

### Intestinal epithelial cell proteins

**Figure 1:**
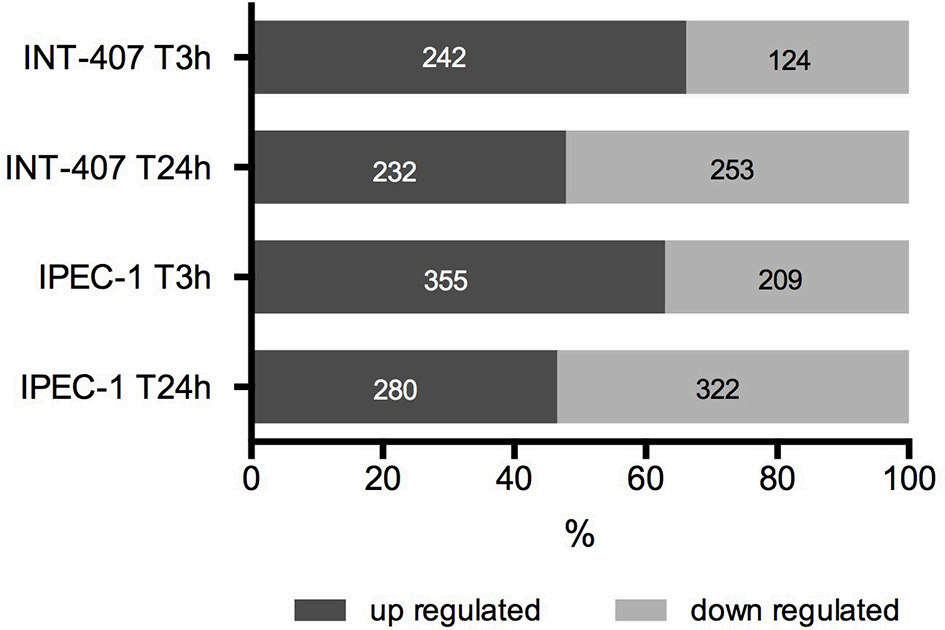
**Percentage and number of up and downregulated proteins in human (INT-407) and porcine (IPEC-1) intestinal epithelial cells at early (T3h) and late (T24h) stages of ***C. jejuni*** infection**.

**Figure 2:**
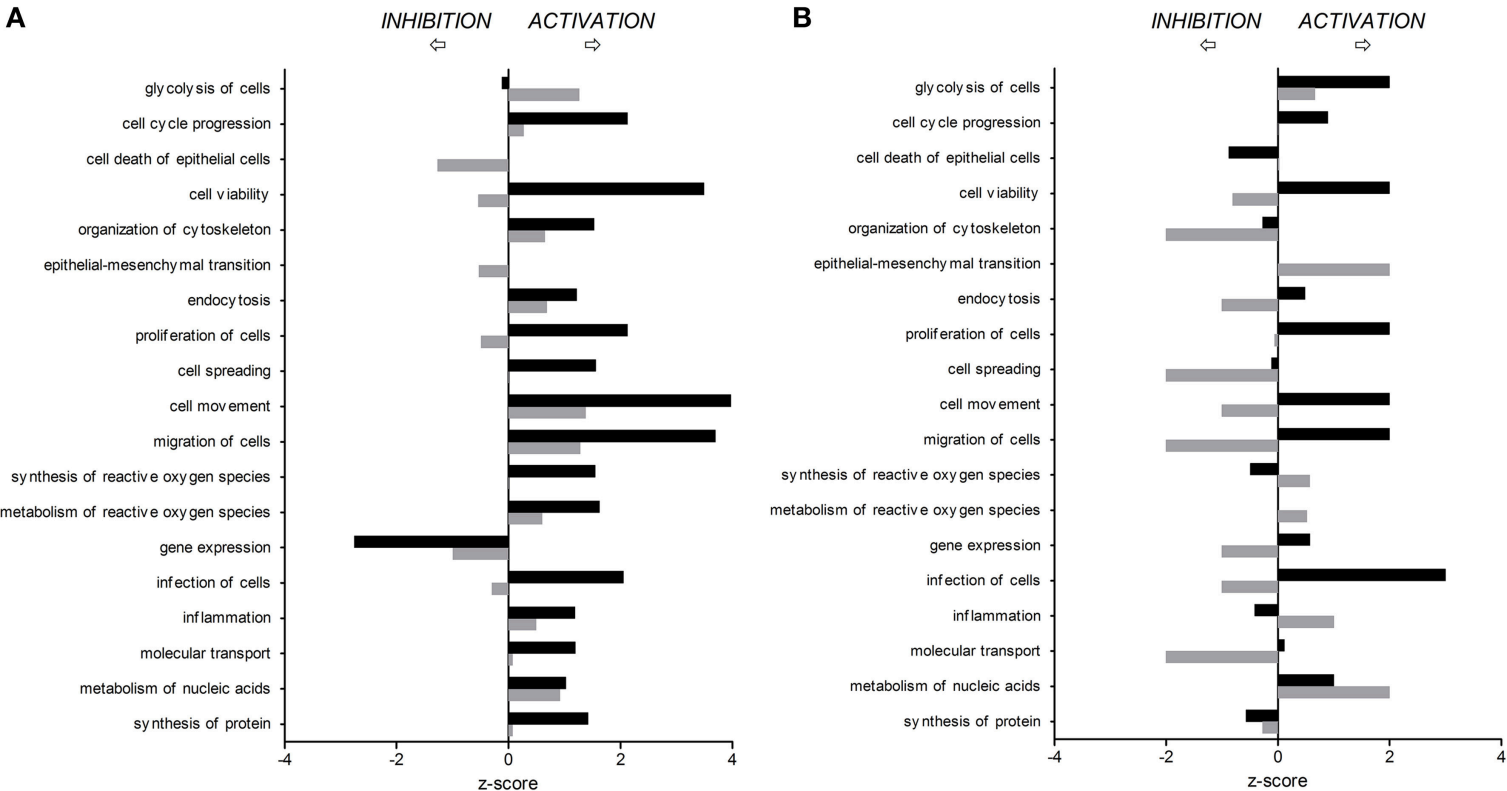
**Predicted activation or inhibition of selected biological functions affected by ***C. jejuni*** infection in human (A)** and porcine **(B)** intestinal epithelial cells 3 h (T3h, black bars) and 24 h (T24h, gray bars) after *in vitro* infection, compared to non-infected cells.

Differentially expressed (DE) proteins were identified both in human and porcine intestinal epithelial cells after *C. jejuni* infection (Figure 1, Table S1). A total of 366 and 485 DE proteins were identified at T3h and T24h time points of infection, respectively, in INT-407 cell line. We then used Ingenuity Pathway Analysis (IPA) to examine the predicted biological effects of the protein expression differences seen between control and infected cells. The results of this analysis (Table S2) predicted a significant activation of the inflammatory response, increased at T3h compared to T24h, and associated to the differential expression of many proteins involved in cell migration and infiltration, cell movement, cytoskeleton organization and promotion of reactive oxygen species (ROS) (Figure 2A). IPA analysis also predicted that infection of cells, molecular transport and synthesis of proteins were functions activated at T3h and reduced at T24h. Similarly, INT-407 infected cells showed increased endocytosis, proliferation and viability at T3h compared to T24h. Cell death as well as gene expression, mainly represented by mRNA expression and translation process, were significantly decreased at early and late time points (Figure 2A, Table S2). Additionally, we found changes in the host energy metabolism at T24h characterized by upregulation of key glycolytic enzymes and transporters for glucose uptake, and downregulation of enzymes participating in the pyruvate metabolism and oxidative phosphorylation (Figure 2A, Table S2). The canonical signaling and metabolic pathways of all differentially expressed proteins in *C. jejuni*-infected cells at 3 and 24 h are shown in Table S3. Table 1 lists the top 10 most significantly enriched pathways. As shown in Table 1, diverse and complex signals related to cell immune/inflammatory transduction pathways, cell death, cell growth and proliferation, and metabolic pathways are involved in the intestinal mucosa after *C. jejuni* infection. The most significant canonical pathway both at early and late time post-infection was eukaryotic initiation factor-2 (eIF2) signaling, which is involved in eukaryotic protein synthesis and plays a central role in antibacterial response. Other pathways involved in translational regulation such as regulation of eIF4 and p70S6K signaling and mTOR signaling, as well as protein ubiquitination and mitochondrial dysfunction were also induced at both times of infection in human cells. Also in INT-407 cells, Ras related nuclear protein (RAN) signaling, caveolar-mediated endocytosis, tRNA charging and granzyme B signaling were induced only at earlier stages of infection, while remodeling of epithelial adherens junction and phagosome maturation were induced only at later times.

**Table 1:** **The top 10 canonical pathways induced by ***Campylobacter jejuni*** in INT-407 and IPEC-1 cells at 3 and 24 h post-infection**.

**Canonical pathways**	**Proteins Up**	**Proteins Down**	**−log (*p*-value)**
**INT-407 T3h**			
EIF2 signaling	47/184 (25.4%)	5/184 (2.6%)	4.78E01
Regulation of eIF4 and p70S6K signaling	22/146 (15.1%)	4/146 (2.7%)	1.84E01
mTOR signaling	21/187 (11.2%)	4/187 (2.2%)	1.47E01
Protein ubiquitination pathway	9/255 (3.5%)	10/255 (3.9%)	6.96E00
RAN signaling	3/16 (18.75%)	3/16 (18.75%)	6.75E00
Caveolar-mediated endocytosis signaling	5/71 (7.1%)	4/71 (5.6%)	5.44E00
Unfolded protein response	4/54 (7.4%)	4/54 (7.4%)	5.42E00
tRNA charging	6/39 (15.4%)	1/39 (2.5%)	5.38E00
Granzyme B signaling	5/16 (31.2%)	0/16 (0%)	5.25E00
Mitochondrial dysfunction	7/171 (4.1%)	6/171 (2.5%)	5.04E00
**INT-407 T24h**			
EIF2 signaling	32/187 (17.1%)	5/187 (2.6%)	2.43E01
Protein ubiquitination pathway	17/259 (6.5%)	15/259 (5.8%)	1.48E01
Regulation of eIF4 and p70S6K signaling	13/150 (8.7%)	6/150 (4%)	9.22E00
Remodeling of epithelial adherens junctions	5/68 (7.3%)	7/68 (10.3%)	7.68E00
Mitochondrial dysfunction	16/188 (8.5%)	3/188 (1.6%)	7.57E00
mTOR signaling	13/193 (9.7%)	6/193 (3.1%)	7.39E00
Phagosome maturation	7/127 (5.5%)	8/127 (6.3%)	7E00
Epithelial adherens junction signaling	9/148 (6.1%)	5/148 (3.4%)	5.4E00
Germ cell-sertoli cell junction signaling	6/163 (3.7%)	8/163 (4.9%)	4.91E00
Unfolded protein response	3/54 (5.6%)	5/54 (9.25%)	4.72E00
**IPEC-1 T3h**			
EIF2 signaling	18/187 (9.6%)	15/187 (8%)	2.03E01
Regulation of eIF4 and p70S6K signaling	15/150 (10%)	8/150 (5.3%)	1.29E01
Integrin signaling	15/208 (7.2%)	9/208 (4.3%)	1.07E01
Protein ubiquitination pathway	18/259 (6.9%)	8/259 (3.1%)	1.02E01
Actin cytoskeleton signaling	12/220 (5.45%)	11/220 (5%)	9.44E00
mTOR signaling	13/193 (6.7%)	7/193 (3.6%)	8.22E00
Germ cell-sertoli cell junction signaling	9/163 (5.5%)	9/163 (5.5%)	7.89E00
Virus entry via endocytic pathways	8/95 (8.4%)	6/95 (6.3%)	7.88E00
Remodeling of epithelial adherens junctions	6/68 (8.8%)	6/68 (8.8%)	7.74E00
ILK signaling	7/187 (3.7%)	12/187 (6.4%)	7.7E00
**IPEC-1 T24h**			
EIF2 signaling	34/187 (18.2%)	18/187 (9.6%)	4.05E01
Regulation of eIF4 and p70S6K signaling	12/150 (8%)	18/150 (12%)	1.9E01
mTOR signaling	10/193 (5.2%)	16/193 (8.3%)	1.22E01
Protein ubiquitination pathway	11/259 (4.3%)	14/259 (5.4%)	8.65E00
Actin cytoskeleton signaling	11/220 (5%)	9/220 (4.1%)	6.62E00
Glycolysis I	9/41 (21.3%)	0/41 (0%)	6.47E00
Caveolar-mediated endocytosis signaling	4/73 (5.5%)	7/73 (9.6%)	6.03E00
ILK signaling	3/187 (1.6%)	14/187 (7.5%)	5.72E00
RAN signaling	1/18 (5.6%)	5/18 (27.8%)	5.65E00
Germ cell-sertoli cell junction signaling	5/163 (3.1%)	10/163 (6.2%)	5.18E00

In the porcine epithelial cells, we found 355 upregulated and 209 downregulated proteins shortly after *C. jejuni* infection (Figure 1). Later, we observed upregulation of 280 molecules and downregulation of 322. The response proteins DISP2 and NAA15 were among the most upregulated molecules, finding higher levels of expression at later stages of the infection (Table S1). When exploring the biological functions altered (Figure 2B, Table S2), we observed a predicted inhibition of apoptosis signaling during early infection. This decreased apoptosis and subsequent increase in cell proliferation was mainly IL-8 and PI3K/AKT mediated (Table S3). In both human and porcine epithelial cell lines, cell survival was activated in early stages, but tended to be inhibited at later stages of infection, as apoptosis increased.

Infection of this porcine epithelial cell line was demonstrated by upregulation of proteasome subunits PSMA1, PSMA2, PSMA5, PSMC5, and PSMD12, as well as other molecules such as CLTA, BSG, IDH1, KPNB1, MAP4, PCBP2, PGM1, RPL10A, RPL12, SF3A1, SF3B2, SNRPD3, TAGLN2, and TFRC (Table S1). However, we did not find upregulation of canonical inflammatory pathways at any time point after infection, and acute phase response was downregulated (Table S3). Actin polymerization was activated at early but inhibited in late stages of infection (Table S3). The endocytic process (membrane ruffling) mediated by beta integrins (e.g., CDC42 and RAC1) was mildly increased at T3h, but inhibited T24h after infection. In IPEC-1 cells, eIF2 signaling pathway was predictably inhibited at early infection, and activated later (Table 1, Table S3). Similarly, cholesterol biosynthesis metabolic process was downregulated at early stages, while it was increased at later stages, thus inhibiting NFκB-mediated transcription of inflammatory mediators. Glycolisis and gluconeogenesis were upregulated 24 h after infection.

### *C. jejuni* proteins

The label-free quantification by SWATH-MS allowed the identification of 53 and 31 *C. jejuni* proteins upon infection of INT-407 (Table S4) and IPEC-1 cells (Table S5). Some of these proteins are related to virulence and others are involved in bacteria survival, metabolism, DNA replication and genetic information processing. The virulence factors expressed by *C. jejuni* in human and porcine intestinal epithelial cells are shown in Table 2. Eight virulence proteins were exclusively detected in INT-407 cells, including two type VI secretion system effectors (pebA and hcP), two adaptive response proteins (the chaperone dnaK and the histidine kinase cheA), the p19 iron acquisition protein, the thiol-peroxidases Tpx that plays a role in oxidative stress protection, and the two-component regulator Cj0355c. Ten *C. jejuni* virulence proteins associated to adherence, adaptive response, oxidative stress and iron acquisition were expressed both in human and porcine intestinal epithelial cell lines. Interestingly the expression of some of these proteins in each cell type was markedly different. For example, at early infection, the flagellin protein flaA, the methyl accepting chemotaxis protein cstIII (*p* < 0.01) and the chaperonin groL (*p* < 0.01) expression was upregulated in INT-407 cells (Table S4). In contrast, flaA and groL expression was downregulated in porcine IPEC-1 cells after *C. jejuni* infection whereas cstIII (*p* = 0.33) remained unchanged (Table S5). Iron captation proteins such as iron deficiency induced protein cfbpA and the ferric enterobactin receptor cfrA expression resulted upregulated at T24h in both cell lines compared to T3h (*p* < 0.001).

**Table 2:** *****Campylobacter jejuni*** virulence factors identified in INT-407 and IPEC-1 infected cells using SWATH-MS label-free**.

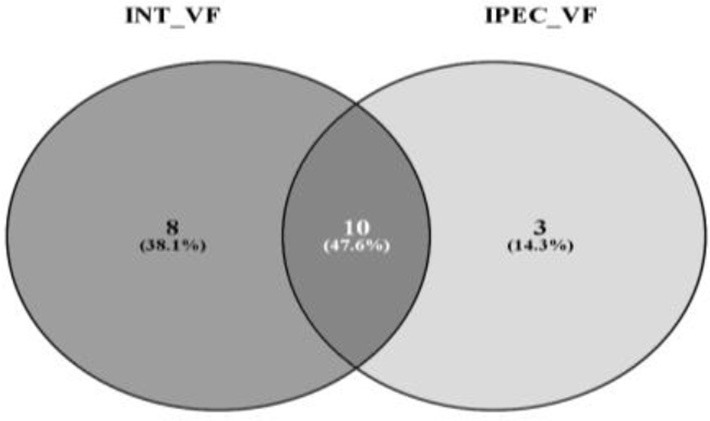				
**Protein**	**Gene**	**Type of function**	**INT-407**	**IPEC-1**
Adhesin pebA	*PebA*	Adherence	Detected	−
Chaperonin GroEL	*groL*	Adherence	Detected	Detected
Methyl-accepting chemotaxis protein CstIII	*cstIII*	Adherence	Detected	Detected
Flagellin FlaA	*flaA*	Motility	Detected	Detected
Chaperone protein DnaK	*dnaK*	Heat shock response	Detected	−
Chemotaxis histidine kinase cheA	*cheA*	Heat shock response	Detected	−
Chaperone protein HtpG	*htpG*	Heat shock response	Detected	Detected
Chemotaxis protein	*N218_03105*	Heat shock response	Detected	−
Peroxidase	*ahpC*	Oxidative stress	Detected	Detected
Periplasmic protein p19	*P19*	Iron captation	Detected	−
Iron deficiency-induced protein A	*cfbpA*	Iron captation	Detected	Detected
Ferric enterobactin receptor cfrA	*cfrA*	Iron captation	Detected	Detected
Superoxide dismutase	*sodB*	Oxidative stress	Detected	Detected
Thiol peroxidase tpx	*tpX*	Oxidative stress	Detected	−
Two-component regulator Cj0355c	*Cj0355c*	Oxidative stress	Detected	−
Putative membrane protein CjaE	*cjaE*	Oxidative stress	Detected	Detected
Type VI secretion system protein	*hcP*	Virulence factor	Detected	−
Peptidoglycan-associated essential protein cjaD	*cjaD*	Virulence factor	Detected	Detected
Thioredoxin	*trxB*	Oxidative stress	−	Detected
Hydrogenase 2 large subunit	*M635_02020*	Oxidoreduction process	−	Detected
Lytic transglycosylase	*mltE*	Cell wal murein synthesis	−	Detected

A relevant number of *C. jejuni* proteins identified in this study were involved in metabolism. Thus, 33 proteins associated to metabolic processes were detected in INT-407 infected cells, 16 of these proteins showed significantly (*p* < 0.05) reduced levels at T24h compared to T3h and only three proteins showed a moderate increase in their levels at 24 h post-infection. This *C. jejuni* metabolic downshift at T24h of infection was not observed in IPEC-1, where from 12 *C. jejuni* identified proteins associated to metabolism, only hisF (*p* < 0.01) and acnB (*p* < 0.05) resulted significantly downregulated after 24 h of infection.

**Figure 3:**
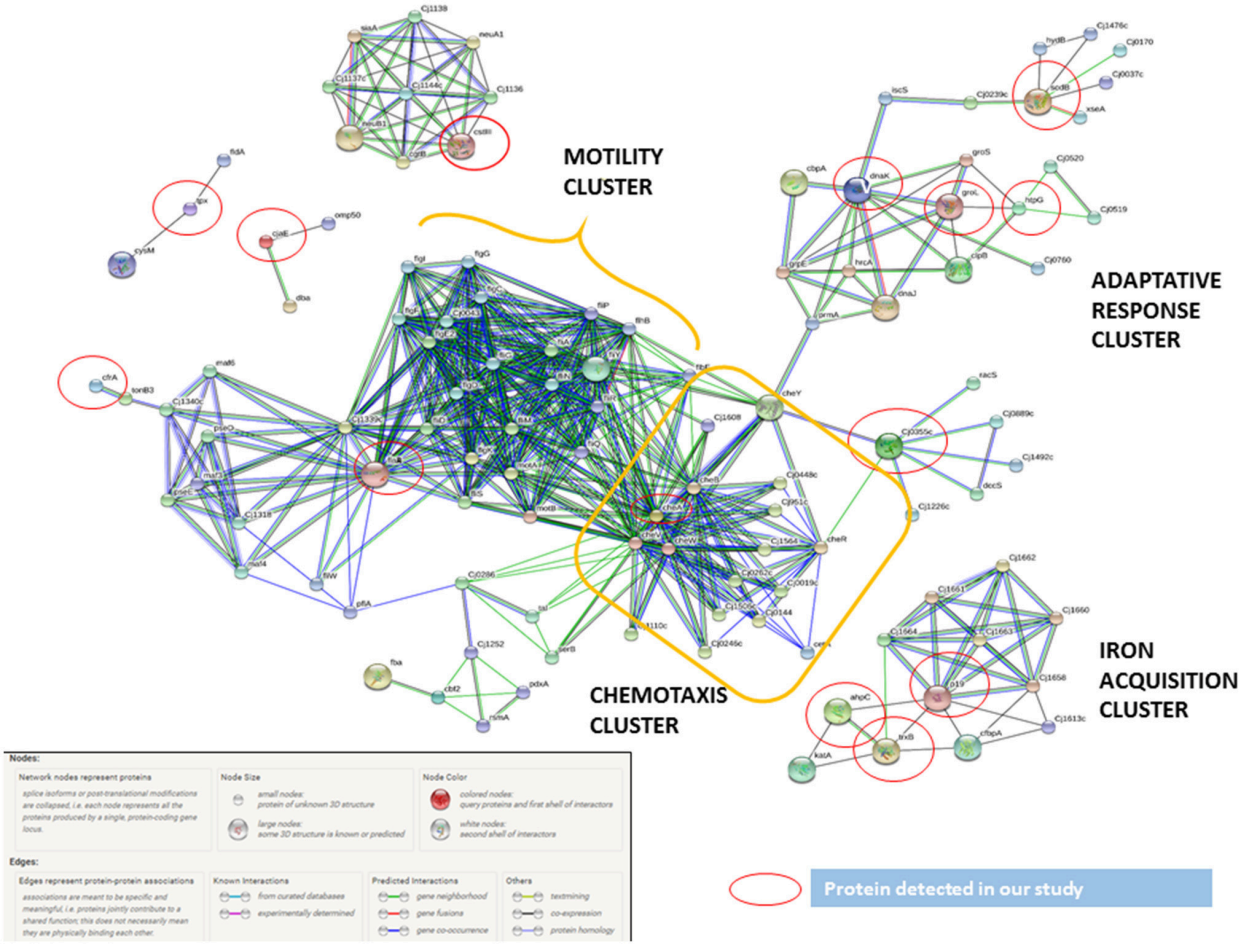
*****C. jejuni*** virulence proteins interaction network**. The network allocated the *C. jejuni* detected proteins in INT-407 cell line (red circles) together with functional partners. The biggest cluster was associated to chemotaxis and related to cluster including all motility proteins. The other proteins included were associated to adaptive response (heat shock and oxidative stress proteins) and iron acquisition proteins.

By using STRING tool we built an enriched model to predict associations among observed *C. jejuni* proteins and functional partners (Figure 3, Table S6). The predicted model organized proteins in different clusters. The main cluster was built around cheA chemotaxis protein, surrounded by 15 functional proteins in total. This “chemotaxis cluster” was closely linked to the “motility cluster,” where the adhesion protein flaB was associated to flagellar proteins such as fliD, fliY, or motA as well as the protein pseE, involved in the glycosylation of the flagella (Figure 3). The heat-shock proteins dnaK and htpG were clustered together and with the chemotaxis protein groL. Finally, two “iron uptake clusters” were conformed. The siderophor cbpA and iron captation protein p19 were joined to the oxidative stress protein ahpC and other functional partners, which include the permease cfbpB, the lipotrotein cj0176c, and the tioredoxin trxB. On the other hand, iron captation protein cfrA was included in other iron uptake cluster, among its partners are included the protein exB1 associated to iron uptake from the host and the protein atpD, involved in the synthesis of ATP, which was detected in our study, although not included as virulence factor.

## Discussion

Despite the fact that *Campylobacter* continues to be the leading cause of bacterial foodborne diarrheal disease in EU (EFSA, 2015), little is known about the molecular mechanisms underlying the intestinal infection caused by bacteria. Much less is known about why *Campylobacter* is pathogenic to humans but acts as commensal bacterium in the gastrointestinal tract of pigs (Horrocks et al., 2009; Bratz et al., 2013). In a previous work we demonstrated that *C. jejuni* adheres preferentially to human intestinal epithelial cells and that this cellular tropism was accompanied by a strong inflammatory response (Aguilar et al., 2014). In this work, using a next generation proteomic approach, we sought to understand better the duality of behavior from commensal to pathogen that *Campylobacter* manifests in the porcine and human host, respectively.

As expected, *C. jejuni* infection prompted an intense inflammatory response in human intestinal cells, mainly characterized by expression of proteins involved in cell spreading, cell movement or cell migration and promotion of reactive oxygen species (ROS). In addition, the eIF2 signaling pathway, involved in inflammatory response, was the most representative canonical pathway, with major activation state at T3h, when bacterial invasion was more intense. eIF2 signaling, critical for stress-induced regulation of translation in eukaryotic cells, is activated by pathogens and is part of a general antibacterial defense system (Shrestha et al., 2012). In agreement with previous studies (Skjolaas et al., 2007; Horrocks et al., 2009; Bratz et al., 2013; Aguilar et al., 2014), we found just a slight inflammatory response in porcine intestinal cells after *Campylobacter* infection, indicating a potential commensal behavior.

*C. jejuni* uses different strategies to enter intestinal epithelial cells such as receptor binding or membrane ruffling (Krause-Gruszczynska et al., 2007; Croinin and Backert, 2012; Eucker and Konkel, 2012). In this work, proteomic analysis highlighted membrane ruffling associated with bacterial entry in INT-407 cells, evidenced by biofunctions related with engulfment and endocytosis, and supported by canonical pathways associated to infection such as caveolar- and clathrin-mediated endocytosis signaling. In porcine IPEC-1 cells, the endocytic process was mildly increased at T3h but inhibited 24 h after infection. The presence of membrane ruffling commonly involves the activation of small Rho family GTPases to induce cellular responses during the infection process (Krause-Gruszczynska et al., 2011). The cell division control protein 42 homolog (CDC42) is a prominent member of this protein family, whose role is to regulate signaling pathways that control cellular functions such as cell migration, endocytosis and cell cycle progression. Here we found upregulation of CDC42 in the early stages of the *C. jejuni* infection in INT-407 cells but downregulation in porcine intestinal epithelial cells, according with the lower levels of bacterial invasion previously observed in the porcine cells (Aguilar et al., 2014). The decrease of cytoskeletal reorganization observed in IPEC-1 cells, probably due to the downregulation of the cytokeratins KRT18/KRT8 and filamin A (FLNA), both activators of the organization and cross linking of actin filaments (Shi et al., 2013), can also be an indicator of reduced cell invasion.

In addition to invasion, pathogenesis of enteric *Campylobacter* infection depends on the ability of bacteria to adhere the intestinal epithelial barrier by adhesins, chemotaxis proteins, and binding proteins (Dasti et al., 2010), causing epithelial damage, loss of cellular function, liberation of electrolytes and finally diarrhea (Everest et al., 1992; Carvalho et al., 2001). In this study, a number of *C. jejuni* proteins related to motility, adhesion and invasion were detected at the time of infection of intestinal epithelial cells. Among these factors, it was included flaA, which is a major component of the *C. jejuni* flagellum filament, involved in motility and secretion (Sulaeman et al., 2012), and a major factor in adherence and invasion of the host cells (Yao et al., 1994). Other adherence and invasion factors found were the periplasmic invading protein pebA (Sulaeman et al., 2012) and the methyl-accepting chemotaxis protein (CstIII) (Hendrixson and DiRita, 2004). The overexpression of all these proteins in INT-407-infected cells at T3h was one of the major insights of this study, showing the success of the *C. jejuni* adhering and invading INT-407 cells in early stages of the infection. In contrast, in IPEC-1 cells, these proteins were either not detected or their expression was downshifted evidencing the existence of clear differences in expression of *C. jejuni* adherence and invasion factors between susceptible and commensal hosts, and confirming the insights stated by Aguilar et al. (2014). However, no marked differences were observed in the expression of other virulence proteins grouped in adaptive response and iron acquisition functions, both required to colonize and invade the host cell (Miller et al., 2009).

Also, a relevant number of proteins and enzymes related to *C. jejuni* metabolic routes were detected in both cell lines, but particularly in INT-407 cells. Our major finding was that a relevant number of metabolism factors resulted downregulated in the INT-407 cells at 24 h after infection. Liu et al. (2012) already described a remodeling of the bacterial proteome after infection of COS-1 cells in which metabolic factors were downshifted. This transition to a stationary stage could presumably associated with the *C. jejuni* adaptation to the intracellular environment.

In conclusion, the present study reveals marked differences between infective and commensal behavior of *C. jejuni* in human and porcine intestinal cell lines. Inflammatory response, endocytosis and cellular stress observed in human INT-407 cells because of the bacterial infection were not evidenced in IPEC-1 cells. These differences in the translational cell response to infection was supported by the different expression program triggered by *C. jejuni* in human and porcine cells which could be a key factor in successful cell invasion of human intestinal epithelial cells. However, the fact that no marked differences in expression of some virulence proteins required to colonize host cells were observed in INT-407 and IPEC-1 cells, suggests that certain host factors, such as cell receptors, should also be responsible for differences in host cell invasion and consequently for differences in the relationships that *Campylobacter* establishes with the different hosts.

## Author contributions

NA, ÁJ, and CA carried out the experimental infections. JD and MV performed the proteomic quantification and analysis. ÁJ, AM, HA, and SZ performed the biological functions analyses and interpretation of results, drafted and edited the manuscript. JG conceived and designed the project, and participated in the interpretation and discussion of the results, as well as in the writing of the manuscript. NA, ÁJ, HA, SZ, CA, MV, AM, JD, and JG read and approved the final manuscript.

## Funding

This work was supported by the Spanish Ministry of Economy and Competitiveness (AGL2014-54089-R). SZ is a postdoctoral researcher supported by the Postdoctoral Trainee Program of the Spanish Ministry of Economy and Competitiveness (FPDI-2013-15619). HA is a postdoctoral researcher supported by the Juan de la Cierva Postdoctoral Trainee Program of the Spanish Ministry of Economy and Competitiveness (FJCI-2014-22877). MV was supported by the Research Plan of the University of the Castilla-La Mancha, Spain.

### Conflict of interest statement

The authors declare that the research was conducted in the absence of any commercial or financial relationships that could be construed as a potential conflict of interest.

## Supplementary material

Table S1 **Differentially expressed proteins in both cell lines**.

Table S2 **Biological functions IPA results in both cell lines**.

Table S3 **Canonical pathways IPA results in both cell lines**.

Table S4 *****C. jejuni*** proteins identified in INT-407 cell line**.

Table S5 *****C. jejuni*** proteins identified in IPEC-1 cell line**.

Table S6 **String proteins information**.
